# Purification and Characterization of WA18, a New Mycocin Produced by *Wickerhamomyces anomalus* Active in Wine Against *Brettanomyces bruxellensis* Spoilage Yeasts

**DOI:** 10.3390/microorganisms9010056

**Published:** 2020-12-28

**Authors:** Francesca Comitini, Alice Agarbati, Laura Canonico, Edoardo Galli, Maurizio Ciani

**Affiliations:** Dipartimento di Scienze della Vita e dell’Ambiente (DiSVA), Università Politecnica delle Marche, Via Brecce Bianche, 60131 Ancona, Italy; a.agarbati@pm.univpm.it (A.A.); l.canonico@univpm.it (L.C.); e.galli@pm.univpm.it (E.G.)

**Keywords:** killer yeasts, killer toxin, *Wickerhamomyces anomalus*, spoilage yeasts, biocontrol, bioactive yeasts

## Abstract

*Wickerhamomyces anomalus* strain 18, isolated from a natural underground cheese ripening pit, secretes a mycocin named WA18 that inhibits wine spoilage yeasts belonging to *Brettanomyces bruxellensis* species, with a broad-spectrum of activity. WA18 was purified, and the purified protein was digested with specific restriction enzymes (lysine K and arginine R cut sites). The LC–MS and LC–MS/MS analysis after enzymatic digestions revealed a molecular weight of 31 kDa. Bioinformatics processing and database research of digested pure killer protein showed 99% identity with a UDP-glycosyltransferase protein. Competitive inhibition assay of killer activity by cell-wall polysaccharides suggests that branched glucans represent the first receptor site of the toxin on the envelope of the sensitive target. The WA18 partially purified crude extract (PPCE) showed high stability of antimicrobial activity at the physicochemical conditions suitable for the winemaking process. Indeed, in wine WA18 was able to counteract *B. bruxellensis* and control the production of ethyl phenols. In addition, the strain WA18 was compatible with *Saccharomyces cerevisiae* in co-culture conditions with a potential application together with commercial starter cultures. These data suggest that WA18 mycocin is a promising biocontrol agent against spoilage yeasts in winemaking, particularly during wine storage.

## 1. Introduction

Yeast killer toxins are defined as antimicrobial proteinaceous compounds able to counteract sensitive yeast strains, although they remain immune to their own toxin effect [1]. Recently, killer yeasts and relative toxins have been reviewed by several authors discussing the molecular mode of action and their potential applications in view of possible use as preservatives in several fields [1,2,3,4,5].

The use of killer yeasts can be considered a promising strategy in the terrestrial primary production for the control of microbial decay [6,7]. Among them, the killer yeasts *Kluyveromyces wickerhamii*, *Wickerhamomyces anomalus* and *Pichia membranifaciens* represent an excellent example and have been extensively studied for their efficacy toward different sensitive spoilage yeasts in the winemaking field.

*W. anomalus* is an ascomycetous heterothallic yeast of the family *Wickerhamomycetaceae* that reproduces asexually by budding and sexually by the formation of hat-shaped ascospores [8]. Strains of this species are present in several environments and have been mainly isolated from fruits and plant materials, cereal grain, maize silage, high-sugar food products, and wine [8]. *W. anomalus* can grow under extreme environmental stress conditions, such as low and high pH, low water activity, high osmotic pressure and anaerobic conditions [9]. *W. anomalus* is classified as a biosafety level 1 organism that is considered safe for healthy individuals, as reported in the online update of EFSA in March 2020 (https://www.efsa.europa.eu/it/efsajournal/pub/6174). For this, in the last days, *W. anomalus* has been studied for its biocontrol property against spoilage yeasts, mainly *Dekkera*/*Brettanomyces* [10,11]. For example, *W. anomalus* NCYC 434 and its killer protein (panomycocin) has been suggested as a potential antifungal agent [12], while it was revealed that Pikt, a small size mycocin from *W. anomalus,* is able to reduce wine contamination in substitution to sulfur dioxide. More recently, de Ullivarri and coworkers [13] evaluated the killer strain *W. anomalus* Cf20 as a potential biocontrol agent active against a broad spectrum of wine yeasts, such as *Pichia guilliermondii*, *P. membranifaciens*, *Brettanomyces bruxellensis* and *Dekkera anomala*. Furthermore, *W. anomalus* strains have been extensively studied for applications against postharvest spoilage molds during table and or wine grape conservation [14,15]. Indeed, *W. anomalus*, similarly to other yeasts as *Metschnikowia* spp., produces a large spectrum of volatile low molecular weight compounds <300 Da (VOCs) characterized by low polarity and high vapor pressure that strongly inhibit the microbial growth [16,17].

The contaminations of *Brettanomyces* yeasts, the imperfect form of sporogenic *Dekkera*, are responsible for the loss of the fruity aromas of the wines and the formation of undesirable olfactory notes called “Brett character” as 4-ethyl phenol from the p-cumaric acid and 4 ethyl guaiacol from ferulic acid. These compounds have been identified as primarily responsible for the appearance of phenol and animal odors such as horse sweat, medicinal, leather, smoky, earthy, burnt plastic and solvent [18,19]. Based on the wide spectrum of action of the mycocins produced by *W. anomalus* and their strong efficacy towards sensitive yeasts, these toxins and the related producer strains are already applied in enology to control the fermentation process. For example, the mycocin KTCf20, produced by *W. anomalus* Cf20 strain, is a highly stable protein at pH values between 3.0 and 5.5 and temperature up to 37 °C with a wide spectrum of action against spoilage yeasts and represent a potential antimicrobial tool in the cellar [13].

In this work, it was investigated *W. anomalus* strain 18 isolated and identified as a mycocin producer during a microbiological investigation on a typical Italian cheese ripened in a peculiar natural underground pit characterized by anoxygenic conditions and low-temperature [20]. The mycocin named WA18 was purified, biochemically characterized, and preliminarily evaluated against *B. bruxellensis* strains under oenological conditions, monitoring the growth and the production of ethyl phenols in wine.

## 2. Materials and Methods

### 2.1. Yeast Strains and Growth Media

*W. anomalus* strain 18 was isolated and identified in previous work [20] during an isolation campaign of autochthonous yeasts in special underground pits of sandstone rock used to ripen a traditional Italian cheese. All sensitive yeast strains belonging to *B. bruxellensis* coming from the Yeast Collection of the Department of Life and Environmental Sciences (DiSVA) of the Polytechnic University of Marche (Italy). DiSVA potential sensitive strains were mainly isolated from vineyard or cellar environments (Table 1).

All strains were cultured in yeast peptone dextrose (YPD) broth containing 10 g L^−1^ yeast extract, 20 g L^−1^ peptone, 20 g L^−1^ glucose, 18 g L^−1^ agar. If required, for assays in liquid, the pH was buffered at 4.2 with 0.1 M citric acid/dibasic sodium phosphate. For killer activity assay, Malt Agar (Biolife, Monza, Italia) supplemented with 30 mg L^−1^ methylene blue-buffered at pH 4.2 was used. All strains were maintained in YPD supplemented with 20% glycerol at −80 °C. The medium used to produce WA18 was YPD-modified (type A) as following: dextrose 15 g L^−1^, yeast extract 10 g L^−1^, malt extract 5 g L^−1^, casein peptone 5 g L^−1^; buffered to pH 4.4 with 0.1 M citrate phosphate buffer.

Vinification trials were carried out using commercial sterile grape must name Folicello (www.folicello.it) supplemented with 10 mg L^−1^ p-cumaric acid as the main phenol’s precursor.

### 2.2. Killer Assay

The killer activity of *W. anomalus* strain 18 was tested against a panel of yeasts by the diffusion plate assay; the plate was seeded with sensitive strain at a final concentration of 10^6^ CFU mL^−1^ in Malt Agar pH 4.2. An exponential culture of *W. anomalus* was spotted, and after 72 h of incubation at 25 °C, strains were designated as sensitive when the spot grew surrounded by a clear zone of inhibition. The assay was repeated in triplicate. The diameter of the inhibition zone was measured with a caliber. Killer activity (KA) was defined as arbitrary units (AU) per mL and was calculated considering the diameter of the inhibition zone in millimeters, where 1 AU is the amount of toxin capable of producing a clear inhibition zone of 12 mm in diameter.

### 2.3. Test of the Killer Activity of Strain 18

To identify the killer activity of *W. anomalus* strain 18 against sensitive *B. bruxellensis* DiSVA 46 and to demonstrate that the mycocin was produced and extracellular secreted, the well-test screening method was performed. An aliquot of *B. bruxellensis* strain from a YPD agar plate culture was suspended in sterile water until a suspension with OD 600 of 0.1 (approximately 10^6^ CFU mL^−1^) was obtained. Inside the Petri dish, 1 mL of the microbial suspension was poured and distributed and 20 mL of Malt Agar at pH 4.2 with 0.1 M citrate-phosphate buffer. Sterile wells with a diameter of 6 mm were placed on still liquid agar. After solidification, the wells were removed with sterile forceps and each space was filled with 70 μL of supernatant filter-sterilized through 0.45 μm pore-size filters. The extracellular killer activity was evaluated by measuring the diameter of the zone of inhibition around the wells [21] after incubation at 25 °C for about five days.

### 2.4. Production and Purification of WA18

To establish the maximum amount of mycocin produced and the corresponding time, *W. anomalus* was cultivated in YPB broth in a 1 L Erlenmeyer flask with a 300 mL working volume (Table 2). During the microbial growth, samples were taken every 2 h to evaluate both cell growth and the diameter of inhibition halo. In the purification trials, *W. anomalus* strain 18 was inoculated at 1 × 10^6^ CFU mL^−1^ in type A medium broth pH 4.2 and incubated at 25 °C for 48 h. The medium contained a limited concentration of glucose to avoid the presence of residual sugars in the concentrated product, which during experiments in wine could represent a nutrition source for sensitive cells. The culture was centrifuged at 14,000× *g* for 10 min, and the crude extract obtained was sterilized by filtration with 0.2 µm PVDF filters (Millipore).

For the purification procedures, 5 L of fermented broth was filter-sterilized (0.45 µm pore size filter) was concentrated by two steps of ultrafiltration: (i) tangential flow in a Bioflex apparatus (Schleicher e Schuell GmbH, Germany) using a membrane with a cutoff of 10.000 Da; (ii) ultrafiltration step using cell stirred apparatus (Amicon^®^, Merk, Roma, Italy) until a final volume of 50 mL. The sample was subsequently dialyzed into a 14,000 Da cutoff capillary tube in 2 L of 0.01 M citrate-phosphate buffer at pH 4.2 overnight stirring. This ultra-filtered and dialyzed sample was used for the further characterization of WA18 mycocin.

The PPCE was purified by size exclusion chromatography (Sephadex G-75) through a 1.5 × 160 mm^2^ Sephadex G-75 column with 0.1 M citric acid/dibasic sodium phosphate pH 4.2 at a flow of 0.25 mL min^−1^. Due to unsatisfactory results, the same amount of sample was loaded in ionic exchange chromatography using a strong cationic SP Sepharose fast flow (GE Health Care) matrix set up in a 15 × 230 mm^2^ column and using as NaCl elution buffer in increasing concentrations from 0.1 to 0.5 M. The flow was set to 1 mL min^−1,^ and 5 mL volume fractions were collected. The active fractions were combined, dialyzed and undergone a second ultrafiltration treatment using a membrane with 50 kDa cutoff. During the purification procedure, the killer activity of fractions and relative protein concentration was quantified by a well-test assay and the Bradford procedure for protein evaluation [22]. Purified WA18 fraction was subsequently analyzed by sodium dodecyl sulfate-polyacrylamide gel electrophoresis (SDS–PAGE) using silver staining technique [23].

### 2.5. Binding of WA18 by Cell-Wall Polysaccharides

The sensitive *B. bruxellensis* type strain DiSVA 46 (10^5^ CFU mL^−1^) was treated with the mycocin (70 AU mL^−1^) in the absence or presence of 9 mg mL^−1^ of the following cell-wall polysaccharides: laminarin (Sigma-Aldrich, Saint Louis, MO, USA), mannan (Sigma-Aldrich, Saint Louis, MO, USA), glucan (Sigma-Aldrich, Saint Louis, MO, USA) and pustulan (Calbiochem). After incubation at 20 °C for 24 h, the cell samples were subjected to a well test to quickly observe the inhibition and to viable cell counts to assess the binding activities of the polysaccharides.

### 2.6. WA18 Sequencing

A high-resolution LC–MS analysis was performed on the purified fraction to obtain the accurate molecular weight of the protein. Subsequently, digestion with two restriction enzymes (Lis-C, lysine cutoff site (K) and trypsin; lysine (K) and arginine (R) cut sites, was carried out (Sigma-Aldrich, USA). The LC–MS and LC–MS/MS analysis of the two enzymatic digests obtained will be carried out to obtain the high-resolution fragmentation spectra related to the peptides.

The bioinformatics processing of the fragmentation spectra related to the peptides obtained from the two triplex digestions was elaborated by means of different research approaches in the NCBI database and of number sequencing to maximize the statistical accuracy parameters related to the identification of the sequences.

### 2.7. Protease Treatment

WA18 purified proteins were subjected to protease treatments. Five hundred microliters of purified toxin (0.017 mg mL^−1^) were mixed with 125 μL of each of the following proteases: trypsin, papain, proteases XIV and XVIII, and pepsin (10 mg mL^−1^) and incubated at 25 °C for 24 h and/or 72 h, as previously described by Comitini et al. [22]. The residual killer activities were evaluated by means of the well-plate assay as described above.

### 2.8. Stability of WA18

For mycocin stability assays, the pH and temperature of two identical PPCE lots (70 AU mL^−1^) already produced and with tested inhibition halo of 30 mm were modified for pH and temperature values and tested after 1.30 h. In particular, pH ranges from 2.5 to 5.75 adjusted with 1 M HCl or 1 M NaOH (with intervals of 0.25 pH points), and temperature intervals from 10 to 75 °C (with intervals of 5 °C) were assayed, respectively. After each treatment, residual killer activity was measured by the well-test method in the plate, comparing the obtained results with untreated PPCE. The reduction was calculated as the percentage of discrepancy in diameter halos from optimal pH and temperature conditions.

### 2.9. Evaluation of Fungicide Activity of PPCE WA18 against Brettanomyces bruxellensis Strains in Wine

The effective antimicrobial activity of WA18 toward *B. bruxellensis* strains, which can occur in wine, was evaluated. The main characters of wine were: SO_2_ free; pH 3.5; ethanol 10% *v/v*. Preliminary, 70 mL of natural red must in 100 mL flask supplemented with 10 mg L^−1^ of p-cumaric acid was inoculated with 1×10^5^ CFU mL^−1^ of 17 different *B. bruxellensis* strains (previously isolated from contaminated wine) and incubated at 25 °C for five months. After this time, only the *Brettanomyces* strains that survived with cell concentrations greater than or equal to 1×10^4^ CFU mL^−1^ were selected. Among them, the *B. bruxellensis* viable cell concentration of final wine was standardized at 3.5 × 10^3^ CFU mL^−1^ using a natural wine SO_2_ free and transferred in tubed of 10 mL containing 9 mL, with or without 100 µL of PPCE of WA18 (70 of AU mL^−1^). The vitality of *B. bruxellensis* potential sensitive strain was evaluated periodically within the first 60 days by viable cell counts (data not shown). After 60 days, wine samples were evaluated for the ethyl phenol and ethyl guaiacol concentration by the solid-phase microextraction (HS-SPME method following the protocol described by Canonico et al. [24].

### 2.10. Co-Culture of W. anomalus 18 with S. cerevisiae Strains

*W. anomalus* pure and mixed cultures (*W. anomalus* 18/*S. cerevisiae* commercial starters QA23, BC, BM45, EC1118 (Lalvin, D&C wine) were set up to exclude the negative influence of killer strain on alcoholic fermentation of some most diffused starter strains. Flasks containing 70 mL of pasteurized commercial red must were aseptically inoculated with the different *S. cerevisiae* cultures to obtain an initial cell density of 5 × 10^6^ CFU mL^−1^ and 1 × 10^6^ CFU mL^−1^ of *W. anomalus* strain 18 (mixed culture). Microfermentations were incubated at 16 °C for 10 days. Enumeration of *W. anomalus* was carried out in Lysin medium since *S. cerevisiae* is not capable of growing in this medium, whereas total yeast count was performed in YPD medium.

## 3. Results

### 3.1. Anti-Brettanomyces Spectrum

To assay, the anti-*Brettanomyces* spectrum and the effectiveness of the mycocin of *W. anomalus* 18, *B. bruxellensis* strains coming from different sources such as grapes, winery or beer were used. *W. anomalus* strain 18 was able to inhibit 62 out of 75 *B. bruxellensis* strains assayed (83%), of which 10 strains were strongly inhibited while 7 strains were only slightly inhibited, as shown in Table 1.

### 3.2. Characterization of WA18

**Figure 1:**
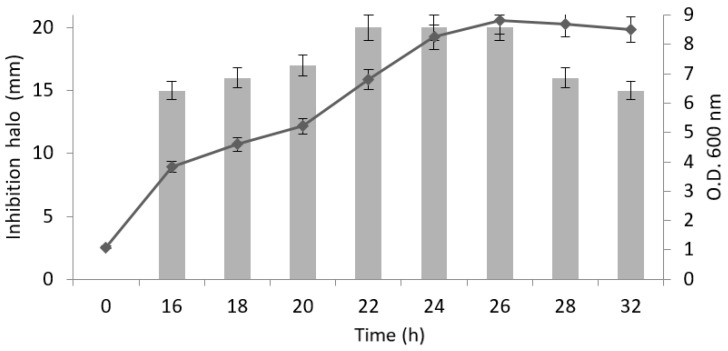
Correlation between *W. anomalus* strain 18 growth evaluated by measuring optical density O.D. 600 nm (line) and production of mycocin (bars) expressed as the diameter of the halo produced against sensitive strain *B. bruxellensis* DiSVA 46. Results were obtained from two experiments carried out separately, and relative standard errors were added.

To further understand the nature of WA18, the production of mycocin was relatively quantified by assigning arbitrary units (AU) monitored during the batch cultivation of the producing strains by means of the well-plate assay (Table 2). In this case, *B. bruxellensis* DiSVA 46 was used as a sensitive strain. Results showed that the production of WA18 mycocin started after 16 h of incubation (beginning of the exponential growth phase) when a visible inhibition halo around the well appeared (Figure 1). Well tests revealed a constant increase in toxin production during exponential growth with a peak during the early stationary phase, until the 32nd h. However, WA18 production was highest and stable between the 22nd and the 26th h (Ø halo = 20 mm).

The killer activity was present at a pH range from 3.25 to 4.50 with pronounced activity at pH 4.00. The highest killer activity was observed at 20 °C temperature and 4.20 pH value (Table 3).

Regarding thermostability, the toxin was stable until 25 °C with a lost 10% of activity starting from 30 °C (after 1 h and half of the incubation) (Table 3). To confirm the proteinaceous nature of WA18, the mycocin was subjected to protease treatments. As expected, the WA18 was sensitive to proteolytic enzymes used, thus confirming the proteinaceous nature of mycocin (data not shown).

With the aim of identifying the first receptor site of WA18 on the envelope of sensitive cells, the competitive inhibition of mycocin by cell-wall polysaccharides was investigated using mannan, laminarin (mainly *β*-1,3-, with a few *β*-1,6-linked glucans), pustulan (*β*-1,6-glucan) and glucan (*β*-1,3- and *β*-1,6-branched glucans). The results show that only glucans were able to competitively inhibit killing action and enhance cell survival. In contrast, mannan, laminarin and pustulan could not rescue the cells, possibly due to their inability to bind WA18 (Table 4).

### 3.3. Production and Purification of WA18

**Figure 2:**
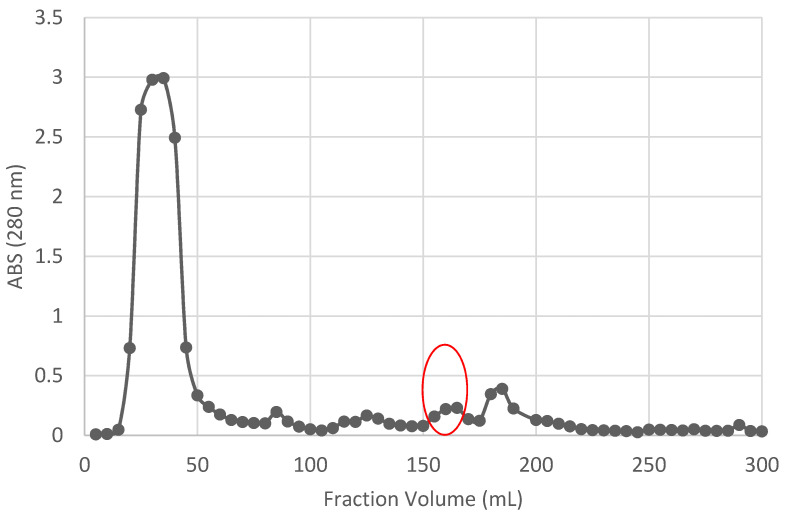
Fractions eluted using SP Sepharose-cation exchange chromatography. Circled peak corresponds to the active fractions (with killer activity).

**Figure 3:**
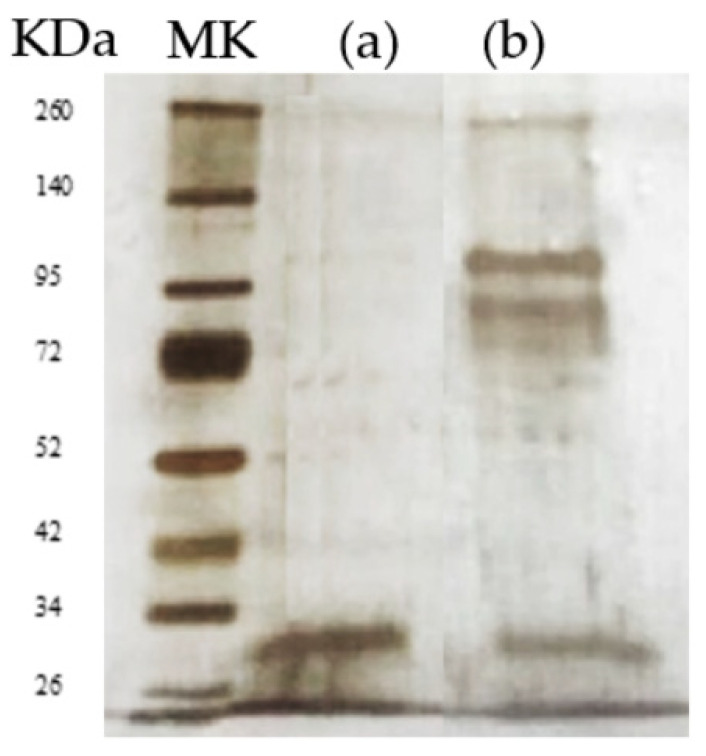
SDS Page of purified WA18 mycocin. MK marker; (**a**) purified fraction; (**b**) SP Sepharose-cation exchange chromatography active fractions.

*W. anomalus* strain 18 was grown in type A medium for 28 h at 20 °C. The broth was then filtered and concentrated to a final volume of approximately 50 mL reaching a 100 × concentrate (PPCE). Due to unsatisfactory results, after several attempts (gel filtration and different ionic exchange matrix), the PPCE was loaded in an SP-Sephadex ionic exchange chromatography (strongly cationic matrix) that revealed the presence of a major peak corresponding to the unbound fraction eluted without salt. The elution with increased concentration of NaCl allowed to separate WA18 mycocin in three fractions where the 280 nm absorbance was higher, the well test showed distinctive inhibition halos, and the relative protein concentration were 67.18, 71.82 and 38.52 mg L^−1^ in fractions #36, #37 and #38, respectively (data not shown). After being tested the positive three fractions separately, they were pooled and loaded on polyacrylamide gel (Figure 3) to observe the corresponding protein bands (Figure 2).

The gel showed the presence of two high molecular weight bands (greater than 72 kDa) and another around 30 kDa. This required a further step before reaching purification; the pool of three active fractions was subjected to a second ultrafiltration step (membrane with cutoff 50 kDa) in which the filtrate (molecular weight less than 50 kDa) was separated from the retentate (molecular weight greater than 50 kDa).

The purification of protein was confirmed by SDS-Page that revealed the presence of a single band with an apparent molecular weight of about 31 kDa (Figure 3). Biochemical steps carried out to purify WA18 killer toxin allowed to obtain a purification yield of about 5% (with 194 purification fold) in a final 500 µl fraction with 27.5 AU mL^−1^, corresponding to 0.017 mg mL^−1^ of protein (Table 2).

**Figure 4:**
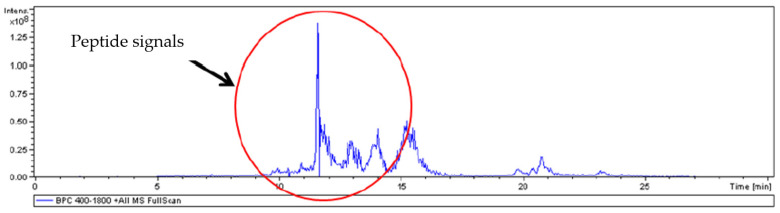
Signal peptide analysis graphic of consensus amino acid sequence of digested WA18 purified mycocin.

The sequencing analysis of purified WA18 (visualized in a single pick after 11 min of chromatography) showed no match with known proteins, using generic databases (Protein Blast NCBI Library), as reported in Figure 4. The peptide sequences detected by the DeNovo approach and selected for statistical accuracy were able to provide important information after a Blast analysis for homology. Sequences due to trypsin fragments and other contaminants were filtered and excluded. The most probable candidate has a primary structure analogous to the UDP-glycosyltransferase protein, also confirming the molecular weight.

### 3.4. Effect of WA18 in Wine

In order to evaluate WA18 antimicrobial properties related to its potential use in winemaking, the antagonistic activity against *B. bruxellensis* strains in wine was investigated. WA18 mycocin (PPCE, AU mL^−1^) was tested in wine in which 9 different *B. bruxellensis* strains (the only survivors after five months in wine) were inoculated in sterile must and grown for 5 months. Wines treated with PPCE WA18 mycocin /700 AU L^−1^ exhibited only a slight increase of phenols concentration after 60 days, around 5%, with the only exception of 212-C strain that showed a large increase of 22.6% and 66.7% of 4 ethyl phenol and 4 ethyl guaiacol, respectively (Table 5). Differently, without the mycocin, a large enhancement of 4 ethyl guaiacol was found; the trends of 4 ethyl phenol were variable and strongly linked to the strain considered. Indeed, in relation to the strains of *B. bruxellensis* 10-B, 14-A, 219A, 25-B and 212-C, the increase varied from a minimum of 24% to a maximum of about 880% of the initial concentration of ethyl phenols.

## 4. Discussion

The application of emerging technologies based on the use of bioactive microorganisms for food and beverages microbial control and preservation is a relevant topic in the wine industry [25,26]. This natural approach can be highly efficient in reducing the growth of spoilage microorganisms as well as the production of undesirable microbial metabolites during processing. As a direct consequence of the proper use of bioactive yeasts, oenologists can easily manage fermentation. Moreover, the production of undesired compounds can be controlled, and the quality of wine can be improved by reducing the use of chemical compounds.

In this work, it was investigated a new mycocin WA18 produced by *W. anomalus*. This killer yeast was isolated in a natural underground pit used for maturing cheeses, a habitat at low-temperature (<12 °C), at a high relative humidity (< 70%) and at high concentrations of CO_2_ (reduced environment) [20]. Probably the extreme environmental conditions to which this yeast has adapted made WA18 mycocin able to resists under winemaking conditions. The wide spectrum of activity showed by WA18 toward *B. bruxellensis* strains is in agreement with the inhibitory spectrum showed by other investigated strains of *W. anomalus* toward *B. bruxellensis*, described in the literature [22,27,28]. Regarding the main biochemical characteristics, WA18 showed an apparent molecular weight of about 31 kDa and exhibited an identity with a glycosyltransferase protein, while glucans were the receptor sites of the cell wall. These results showed some similarities and several differences with the other *W. anomalus* characterized mycocins. Indeed, Pikt from *W. anomalus* showed a ubiquitin-like peptide structure with a molecular mass of approximately 8 kDa and that it selectively interacts with *β*-1,6 glucans [29] while the mycocin KTCf20 secreted by the strain *W. anomalus* Cf20 bind to *β*-1,3 and *β*-1,6 glucans of the cell wall of sensitive strains [13]. Moreover, the results of the thermostability and pH range of activity of WA18 were very similar to other mycocins produced by *W. anomalus,* which showed activity and stability at acidic pH (2–4.5) and temperature lower than 35 °C [7,13,22,30].

Regarding the biochemical purification process allowed to purify WA18 mycocin, the approach used here (ultrafiltration, ion-exchange chromatography and double ultrafiltration steps using different cutoff membranes) was also applied by Villalba et al. [31] during *Saccharomyces eubayanus* mycocin (SeKT) purification but using affinity chromatography with pustulans as a pre-purification strategy. On the contrary, de Ullivarri et al. [13] carried out toxin with only one-step of ultrafiltration by using 50 kDa membrane and exclusion chromatography.

Furthermore, the in vitro binding affinity test to cell wall polysaccharides revealed that WA18 mycocin binds branched glucans on the cell wall of sensitive yeasts. The binding to these types of polysaccharides also has been widely reported [12,13,32]. In addition, the thermo-sensitivity of WA18 is similar to other studied *W. anomalus* yeast toxins [7,12,33].

WA18 purified chromatography fraction was subjected to LC–MS/MS analysis, and results allowed a BLAST-p identification as the primary sequence of UDP-glycosyltransferase protein. In addition, Cecarini et al. [34] performed the same analysis for 140 kDa WaF17.12 mycocin produced by *W. anomalous* detected five peptide fractions described as the β-glucosidase enzyme.

From the applied point of view, the null effect of this mycocin against *S. cerevisiae* starter strains tested, represent a positive opportunity for its use in mixed culture, during industrial processes. Furthermore, the antimicrobial activity trials carried out in wine with a WA18 concentration of 700 UA L^−1^ showed that the toxin was able to control the growth of *B. bruxellensis* strains and its production of ethyl phenols. Similar to SeKT from *S. eubayanus* [31], WA18 mycocin reduced the levels of volatile phenols produced by *B. bruxellensis* strains after the time of evaluation of 60 days, showing a reduction of about 10-fold in comparison with samples without the presence of mycocin.

Based on traditional knowledge as well as recent scientific evidence, killer yeasts are one of the most important players in food biotechnology to control *Brettanomyces* yeast in winemaking. Indeed, *Brettanomyces*/*Dekkera* spoilage yeasts affect the wine marketability for red and white wines and also for the sparkling industry [11,35].

In this context, the mycocin WA18 could have good potential as a biocontrol agent active against *Brettanomyces* spp. since the broad-spectrum of activity is shown.

## Figures and Tables

**Table 1:** List of *Brettanomyces* strains used and sources from which they were isolated. In the third column, the symbols report the killer effect of mycocin WA18 on the related sensitive strains. Legend: ++ strong killer action (diameter of halo > 15 mm), + evident killer action (diameter of halo between 14 and 10 mm), +/− weak killer action (diameter of halo < 10 mm and not persistent), − no killer action.

Sensitive Yeast Codes	Isolation Source	Killer Activity
Type strain DiSVA 46	Beer	+
20	Winery environments	++
21-A4	Winery environments	+
21-B1	Winery environments	+
21-C2	Winery environments	+
25-A	Winery environments	+
25-B	Winery environments	+
14-A	Winery environments	+
14-B2	Winery environments	+
14-C	Winery environments	+
10-A	Winery environments	+
10-A1	Winery environments	−
10-B	Winery environments	+
Disva 48	Tea-beer	−
10-C	Winery environments	−
5-6 A	Winery environments	+
5-6 B	Winery environments	+
5-6 C	Winery environments	+
212-C	Winery equipment (connection valves)	+
212-D	Winery equipment (connection valves)	+
219-A	Winery equipment (connection valves)	+
1.M	Red wine (Montepulciano)	+/−
4.M	Red wine (Montepulciano)	++
5.M	Red wine (Montepulciano)	+/−
6.M	Red wine (Montepulciano)	−
11.M	Red wine (Montepulciano)	−
12.M	Red wine (Montepulciano)	+/−
Disva 79	Grape must	++
PSTA 11	Wine	+
EB1G	Montepulciano Wine	++
EB1p	Montepulciano Wine	++
2.3	Corrosol fruit (Cameroon)	+
21.4	Cocoa beans (Cameroon)	−
21.6	Cocoa beans (Cameroon)	−
21.1t1	Cocoa beans (Cameroon)	−
Fi6	Tuscany winery	+
Fi10	Tuscany winery	++
Fi14	Tuscany winery	+
Fi17	Tuscany winery	+
Fi22	Tuscany winery	+
Fi32	Tuscany winery	+
Fi38	Tuscany winery	++
Fi43	Tuscany winery	+/−
126	Chianti Wine	+
113	Chianti Wine	+
129	Chianti Wine	+
130	Chianti Wine	+/−
124	Chianti Wine	+/−
212c	Chianti Wine	+
116	Chianti Wine	+
34	Chianti Wine	+
69	Chianti Wine	−
111	Chianti Wine	+
107	Chianti Wine	−
117	Chianti Wine	−
1189	Winery (fermentation tank)	+
1194	Winery (fermentation tank)	++
1191	Winery (fermentation tank)	++
212d	Winery (fermentation tank)	+
121	Winery (fermentation tank)	+
118	Winery (fermentation tank)	+
106	Winery (fermentation tank)	+/−
1190	Winery (fermentation tank)	++
94	Winery (fermentation tank)	−
90	Winery (fermentation tank)	−
G1	Craft Belgium Gueze beer	+
G2	Craft Belgium Gueze beer	+
G3	Craft Belgium Gueze beer	+
G4	Craft Belgium Gueze beer	+
G5	Craft Belgium Gueze beer	+
G6	Craft Belgium Gueze beer	+
G7	Craft Belgium Gueze beer	+
G8	Craft Belgium Gueze beer	+
G9	Craft Belgium Gueze beer	+
G10	Craft Belgium Gueze beer	+

**Table 2:** Purification of WA18 from *W. anomalus*.

	Total Volume (mL)	Total Protein (mg)	Activity (AU mL^−1^)	Total Activity (AU)	Specific Activity (AU mg^−1^)	Purification (fold)	Yield (%)
Culture broth	5000	595	1	5000	8.4	1	100
Ultrafiltration 10 kDa cutoff PPCE (partially purified crude extract)	50	7.45	70	3500	289.3	34.44	70
Ionic exchange chromatography (SP Sepharose) 10-fold concentrated	15	1.4	104	1560	1114.3	132.64	31.2
Second step of ultrafiltration 50 kDa cutoff	8.3	0.14	27.5	228.25	1630.36	194	4.57

**Table 3:** Influence of pH and temperature on WA18 killer activity.

pH	WA18 Killer Activity Reduction * (%)	Temperature (°C)	WA18 Killer Activity Reduction * (%)
2.50	100	10	0
2.75	92	15	0
3.00	75	20	0
3.25	0	25	0
3.50	0	30	10
3.75	0	35	20
4.00	0	40	30
4.25	0	45	45
4.50	50	50	50
4.75	85	55	85
5.00	90	60	90
5.25	100	65	100
5.50	100	70	100
5.75	100	75	100

* 100 represents the optimal values.

**Table 4:** Evaluation of WA18 killer activity after binding by cell-wall polysaccharides by well test assay against sensitive strain *B. bruxellensis* DiSVA 46.

Cell-Wall Polysaccharides	Halo Diameter (mm)	
Laminarin (L)	27	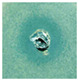
Pustulan (P)	26	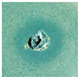
Mannans (M)	28	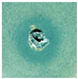
Glucans (G)	0	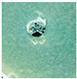
WA18 crude extract (+)	30	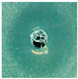

**Table 5:** Effect of WA18 mycocin using partially concentrated crude extract (AU 700 L^−1^ in the wine samples) on ethyl phenol and ethyl guaiacol production by different *B. bruxellensis* strains after 60 days of incubation.

Sensitive Strains	4 Ethyl Phenol (µg/L)							4 Ethyl Guaiacol (µg/L)						
	Initial	w/o WA18	Δ (w/o WA18-initial)	Δ%	WA18	Δ (WA18-initial)	Δ%	Initial	w/o WA18	Δ (w/o WA18-initial)	Δ%	WA18	Δ (WA18-initial)	Δ%
10-B	593	739	146	24.62	633	40	6.75	58	146	88	151.72	70	12	20.69
14-A	368	876	508	138.04	399	31	8.42	44	212	168	381.82	47	3	6.82
219A	410	626	216	52.68	421	11	2.68	59	99	40	67.80	64	5	8.47
25-B	99	972	873	881.82	100	1	1.01	105	222	117	111.43	109	4	3.81
212-C	531	886	355	66.85	651	120	22.60	21	195	174	828.57	35	14	66.67
DiSVA 48	517	532	15	2.90	554	37	7.16	10	101	91	910.00	10	0	0.00
21.6	601	619	18	3.00	606	5	0.83	19	131	112	589.47	24	5	26.32
1191	520	526	6	1.15	519	−1	−0.19	29	116	87	300.00	32	3	10.34
5-6A	421	430	9	2.14	463	42	9.98	65	103	38	58.46	63	−2	−3.08

The initial values are referred to as ethyl phenols concentration after 5 months of fermentation of a sterile red grape must be inoculated with each strain. Δ and Δ% symbols indicate the differences in ethyl phenols content concentration between initial and after 60 days, with and without (w/o) WA18 mycocin.

## Data Availability

Data is contained within the article.
